# Genomic analysis reveals RhoC as a potential marker in hepatocellular carcinoma with poor prognosis

**DOI:** 10.1038/sj.bjc.6601749

**Published:** 2004-05-11

**Authors:** W Wang, L-Y Yang, G-W Huang, W-Q Lu, Z-L Yang, J-Q Yang, H-L Liu

**Affiliations:** 1 Liver Cancer Laboratory, Department of Surgery, Xiangya Hospital, Central South University, 87 Xiangya road, Changsha, Hunan Province 410008, China

**Keywords:** hepatocellular carcinoma, invasion and metastasis, prognostic marker, RhoC, cDNA microarray

## Abstract

Hepatocellular carcinoma (HCC) is one of the most malignant human tumours because of its high incidence of metastasis. The mechanisms underlying the metastasis of HCC, however, remain poorly understood. In this study, we performed cDNA microarray analysis to profile gene expression patterns in two subtypes of HCC, solitary large HCC (SLHCC) and nodular HCC (NHCC), which differ significantly in the incidence of metastasis. Among 668 genes that were differentially expressed, we focused on RhoC, whose expression was significantly decreased in SLHCC compared to NHCC. The expression of RhoC in HCC and pericarcinomatous liver tissues (PCLT) was analysed at both the mRNA and protein levels by reverse transcription–polymerase chain reaction (RT–PCR) and Western blotting. In addition, immunohistochemistry was also performed on 94 cases of HCC with follow-up information. Collectively, our data indicate that the expression of RhoC significantly increased in HCC compared to PCLT; extrahepatic metastatic lesions expressed significantly higher levels of RhoC than the corresponding intrahepatic HCC tissues. There is a highly significant correlation of the RhoC expression levels with tumour vein invasion, number of tumour nodes and the status of differentiation. Significantly, the HCC patients with RhoC-positive expression had shorter survival than those with RhoC-negative expression. Together, our findings suggest a strong correlation between the expression of RhoC and HCC metastasis, implicating RhoC as a potential prognosis marker and therapeutic target for HCC.

Hepatocellular carcinoma (HCC) is one of the most common malignancies in Asia and Africa (Murray and Lopez, 1997; Akriviadis *et al*, 1998), especially in China. It is responsible for approximately one million deaths each year, predominantly in the developing countries (Schafer and Sorrell, 1999). During the past decade, hepatic resection for HCC has evolved into a safe procedure with low operative mortality (Makuuchi *et al*, 1998; Fan *et al*, 1999). However, the long-term survival remains unsatisfactory because of a high incidence of recurrence and metastasis after hepatic resection, with a 5-year actuarial recurrence rate of 75–100% reported in the literature (Poon *et al*, 2000). Most recurrence was attributed to invasion and metastasis of HCC, so the elucidation of prognostic factors reflecting invasion and metastasis not only provides guidance in the choice of treatments but also allows better prognostic counselling.

It has been generally accepted that the invasive and metastatic potential of HCC was mostly attributed to the individual clinicopathological and biological characteristics. The diversity of biological characteristics determines the different invasive and metastatic potential of HCC (Okuda, 1997). Based on this diversity, in our institute, HCC was distinct phenotypically to: solitary large hepatocellular carcinoma (SLHCC, diameter >5 cm, and one node), which often grows expansively with capsule or pseudocapsule formation and has a favourable biological behaviour, nodular hepatocellular carcinoma (NHCC, node number ⩾2) and small hepatocellular carcinoma (SHCC, diameter ⩽5 cm). Our previous study found that SLHCC, although it shared large size, had long-term survival after hepatic resection compared to NHCC, which implied different invasive and metastatic potential between SLHCC and NHCC. However, further investigation of different invasive and metastatic potential between them was anticipated.

RhoC-GTPase, a member of the Ras superfamily of small guanosine triphosphatases (GTPases), shuttles between inactive GDP- and active GTP-bound form and exhibits intrinsic GTPase activities. Activation of Rho protein leads to the assembly of the actin–myosin contractile filaments into focal adhesion complexes that lead to cell polarity and facilitate motility (Nobes and Hall, 1995; Kimura *et al*, 1996; Leung *et al*, 1996). Recently, emerging data suggest that RhoC gene may have a potential role in carcinogenesis and metastasis of tumour cells (Itoh *et al*, 1999; Clark *et al*, 2000). The expression level of RhoC in metastatic region of pancreatic cancer has been previously reported to be higher than primary lesion (Suwa *et al*, 1998). In addition, a genomic-wide analysis of gene expression revealed that RhoC gene was involved in vascular invasiveness of HCC (Okabe *et al*, 2001).

To gain a better understanding of the difference in invasion and metastasis between SLHCC and NHCC, we employed cDNA microarray technology to profile gene-expression patterns in these two subtypes of HCC. Among 668 genes that were differentially expressed between SLHCC and NHCC, *RhoC* gene was selected for further study because of its potential association with tumour metastasis.

## MATERIALS AND METHODS

### Tissue specimens

The study protocol was approved by the Ethics Committee of the Central South University. Fresh samples of HCC tissues and pericarcinomatous liver tissues (PCLT, 1 cm away from the carcinoma) were obtained from 25 (22 male and 3 female) patients with hepatocellular carcinoma who underwent hepatoectomy at Xiangya Hospital of Central South University (CSU). The specimens were immediately flash frozen in liquid nitrogen and stored at −80°C for RT–PCR and Western blotting. The median age of the patients was 53 years, ranging from 28 to 73 years. All specimens obtained from hepatic resection were confirmed by pathological examination and clinicopathological parameters, such as tumour diameter, number of tumour node, tumour capsule, histopathological classification, vein invasion and extrahepatic metastatic lesion. On this basis, the total number of cases could be divided into three groups (SLHCC, *n*=7; NHCC, *n*=15; SHCC, *n*=3).

### RNA isolation

Total RNA was extracted from frozen tissue specimens (30–100 mg) using TRIZOL (GIBCO BRL, Gaithersburg, USA) reagent, according to the instructions provided by the manufacturer. The quality was checked in 1% agarose gels and the concentration was measured using an ultraviolet Spectrophotometer (Biochrom Ltd, Cambridge, England) (Suwa *et al*, 1998). The UV wavelength was adjusted at 260 nm.

### cDNA microarray

cDNA microarray was carried out on 22 cases (7 SLHCC and 15 NHCC) of HCC specimens. Examination of quality and quantity for individual RNA was performed, and all RNA samples were eligible. As we investigated the differentially expressed genes between the SLHCC group and NHCC group, the individual RNA specimens in the same group were mixed equally to obtain 30 *μ*g total RNA, which was reversely transcribed using reverse transcriptase (GIBCO BRL, Gaithersburg, USA). The mixed RNA samples from SLHCC were labelled with Cy-5 and those from NHCC were labelled with Cy-3. After incubation at 42°C for 2 h, the reactions of the two samples (one with Cy-3 and other with Cy-5) were mixed and purified by using a filtration spin column (Promega, Madison, USA). The whole volume of the purified probe was denatured at 95°C for 2 min and was applied to the cDNA microarray slide containing 8464 genes, which was provided by United Gene Holdings (Biostar Genechip Inc., Shanghai, China). The control spots of non-human origin in cDNA microarray slide include the U2 RNA gene, Hepatitis C Virus coat protein gene and spotting solution alone without DNA. After hybridisation at 42°C for 18 h, the slide was washed for 10 min each in solutions of 2 × SCC and 0.2% SDS, 0.1% × SCC and 0.2% SDS and 0.1% × SCC. Finally, the cDNA microarray slide was scanned with a Scan Array 4000 Standard Biochip Scanning System (Packard Biochip Technologies, VA, USA) at two wavelengths to detect the emission of both Cy5 and Cy3. The acquired image was analysed using ImaGene 3.0 software (Bio Discovery, Los Angeles, USA). The intensities of each spot at the two wavelengths represent the quantity of Cy5 and Cy3, respectively, hybridised to each spot. Ratios of Cy5 to Cy3 were computed for each location. The overall intensities were normalised with a correction coefficient obtained using the ratio of 64 housekeeping genes. Genes were identified as differentially expressed if the absolute value of the natural logarithm of the ratios was >0.69. To minimise artefacts arising from low expression values, only genes with raw intensity values for both Cy5 and Cy3 of >800 counts were selected for differential analysis (Li *et al*, 2002).

### Reverse transcription and polymerase chain reaction (RT–PCR)

Total RNA (2 *μ*g) was reversely transcribed in a final 25 *μ*l reaction volume at 37°C for 1 h by using 200 U M-MULV reverse transcriptase (Promega, Madison, USA). PCR amplification was performed in a final volume of 50 *μ*l containing 5 *μ*l first-strand cDNA solution, 2 U Taq polymerase (Sangon, Shanghai, China), 5 *μ*l 10 × PCR reaction buffer, 10 *μ*M of dNTP (Sangon, Shanghai, China) and 10 pmol of each 3′ and 5′ sequence-specific oligonucleotide primer (Sangon, Shanghai, China) for RhoC and *β*_2_-microglobulin gene (positive control). The primer sequences were as follows: human RhoC, 5′-TCCTCATCGTCTTCAGCAAG-3′ (forward), 5′-CTGCAATCCGAAAGAAGCTG-3′ (reverse); and human *β*_2_-microglobulin, 5′-ACCCCCACTGAAAAAGATGA-3′ (forward), 5′-ATCTTCAAACCTCCATGATG-3′ (reverse). The amplification was performed on a DNA Thermal cycler (Perkin-Elmer, Shelton, USA) with an initial denaturation at 94°C for 3 min, followed by 32 cycles of denaturation at 94°C for 40 s, annealing at 56°C for 30 s and extension at 72°C for 1 min, and a final extension at 72°C for 10 min. PCR products were electrophoresed on 1.7% agarose gels; the bands representing amplified products were visualised using ethidium bromide during the exposure to a UV transilluminator. The density of the bands on the gel was quantified by densitometric analysis. RhoC gene expression was presented by the relative intensity of the PCR product bands from target sequences to that from the *β*_2_-microglobulin gene (Suwa *et al*, 1998).

### SDS–PAGE and Western blotting

Tissues from HCC and PCLT were lysed in a lysis buffer; 20 mM Tris-HCl pH=7.4, 10 mM NaCl, 1 mM EDTA pH=8.0, 1 mM MgCl_2_, 1% NP-40, 0.1% SDS, 0.01% PMSF (Sigma, St Louis, MO, USA) and protease-inhibitor (Promega, Madison, USA). The lysates were centrifuged at 13 000 **g** for 20 min at 4°C, and the supernatants were stored at −80°C. Extracts equivalent to 100 *μ*g of total protein were separated by stacking gel (3.5%) and SDS–PAGE (12.5%) separating gel with Tris-glycine system at 200 V for 1 h. Blotting was performed to polyvinylidene fluoride membrane (Sigma, St Louis, MO, USA) at 100 V for 1 h in a tank of transfer buffer (48 mM Tris-HCl, 192 mM glycine, 20% methanol pH=8.4). The membranes were blocked in 4% nonfat dry milk in PBS containing 0.1% Tween-20 for 1 h at room temperature. Then, the membranes were incubated with primary antibody (goat anti-human RhoC polyclonal antibody, Santa Cruz, CA, USA, diluted at 1 : 500) for 1 h at 37°C. After washing, the membranes were incubated with a 1 : 1000 dilution of horseradish peroxidase-linked rabbit anti-goat antibody (Santa Cruz, CA, USA) for 30 min at 37°C. Then the membranes were washed and treated with Western blotting luminal reagent (Santa Cruz, CA, USA) to visualise the bands; the results were obtained on Kodak film and quantified by densitometry (Beckman, South Pasadena, Canada) (Abraham *et al*, 2001).

### Immunohistochemistry

**Figure 1:**
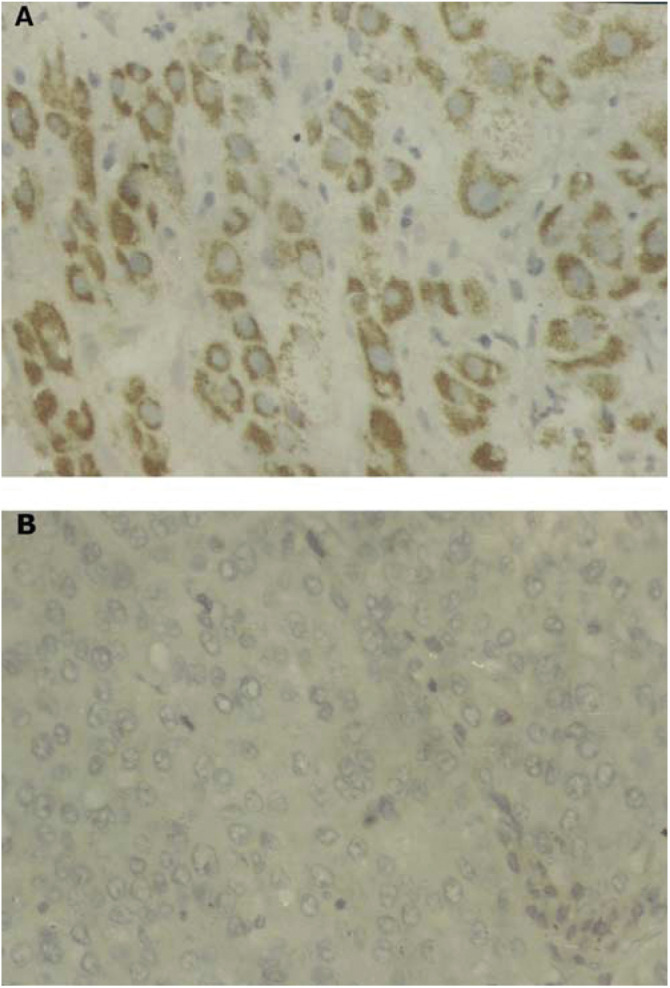
(**A**) Typical staining for positive cytoplastic RhoC expression in an HCC (original magnification × 400). (**B**) Typical staining for negative cytoplastic RhoC expression in an HCC (original magnification × 400).

A total of 94 HCC specimens, including the 25 cases of HCC fresh specimens (used for RT–PCR and Western blotting), were evaluated for immunohistochemistry. All the specimens were collected from Xiangya Hospital of Central South University between 1994 and 2002. Follow-up data were obtained following operation for all patients; the end point of follow-up was set at the patient's death. In brief, tissue sections of 4 *μ*m thick were cut and baked at 60°C for 2 h, deparaffinised in xylene and rehydrated through graded ethanol. Next, 3% hydrogen peroxide was applied to block the endogenous peroxidases for 20 min and sections were subjected to heat-induced antigen retrieval in 0.01 M citrate buffer (pH=6.0). The sections were incubated with normal goat serum to reduce nonspecific binding. Then, they were incubated at 37°C for 1 h with specific antibodies (goat anti-human RhoC polyclonal antibody, Santa Cruz, CA, USA) used at a 1 : 100 dilution. The second antibody was applied for 45 min at 37°C. The streptavidin–biotin–peroxidase complex (SABC) tertiary system (Boster, Wuhan, China) was used according to the manufacturer's instruction for 20 min at room temperature. The tissues were visualised by applying 3,3-diaminobenzidine tetrahydrochloride (DAB) for 3 min. Sections were counterstained using haematoxylin, dehydrated through gradient alcohols and mounted for viewing. Negative controls were carried out by omitting the primary antibody, whereas RhoC overexpression confirmed by Western blotting was used as positive controls. The intensity of cytoplasmic staining was scored as 0 to 3+ by comparison to the positive controls. The scoring system has been previously validated (Kleer *et al*, 2002). Diffuse, moderate to strong cytoplasmic staining characterised RhoC-positive expression (scores 2+ and 3+). RhoC-negative expressions were devoid of any cytoplasmic staining or contained faint, equivocal staining (scores 0 and 1+, Figure 1

)

### Statistical analysis

Statistical analysis was performed using the SPSS (version 11.0, Chicago, IL, USA). The Fisher's exact test was performed to access differences in clinicopathological parameters between SLHCC and NHCC. Spearman's correlation coefficient was used to examine the relationship between expression levels of RhoC mRNA and protein. The nonparametric Mann–Whitney test was monitored to evaluate the differences in the expression of RhoC between groups, and also to analyse the correlation between the expression of RhoC and clinicopathological variables. Postoperative survival was analysed by the log-rank test. Differences were considered significant when *P*<0.05.

## RESULTS

### Clinical and pathological characterisation of SLHCC and NHCC

According to the criteria described in the introduction, there were seven cases of SLHCC and 15 cases of NHCC among the total 25 cases of HCC studied. Most of the SLHCC cases were differentiated at I–II (five out of seven, 71%), whereas considerably fewer cases of NHCC (2 out of 15, 13%) exhibited the same level of differentiation. In addition, there was a substantial difference in tumour vein invasion with a significantly higher percent in NHCC than in SLHCC (one out of seven, 14% SLHCC *vs* 11 out of 15, 73% NHCC). There were five cases of SLHCC, but only three cases of NHCC, which exhibited capsulation (5 out of 7, 71% SLHCC *vs* 12 out of 15, 20% NHCC) also. The distribution of sex, liver cirrhosis in each group showed no significant differences (Table 1

**Table 1:** Clinicopathological differences between SLHCC and NHCC

**Variables**	**SLHCC (*n*)**	**NHCC (*n*)**	**Fisher's test**
*Sex*			
Male	6	14	*P*=1.00
Female	1	1	
			
*Liver cirrhosis*			
Present	5	6	*P*=0.36
Absent	2	9	
			
*Cell differentiation*			
I–II	5	2	*P*=0.01
III–IV	2	13	
			
*Capsule formation*			
Present	5	3	*P*=0.02
Absent	2	12	
			
*Vein invasion*			
Present	1	11	*P*=0.02
Absent	6	4	

).

### Evaluation of invasiveness score for HCC

Invasiveness score (IS) was applied to evaluate the invasive and metastatic abilities of SLHCC and NHCC (El-Assal *et al*, 1997). Three cases of SLHCC (three out of seven) and three cases of NHCC (three out of 15) scored IS⩽1 (low invasive); three cases of SLHCC (three out of seven) and five cases NHCC (five out of 15) scored 2⩽IS⩽4 (moderate invasive); and one case of SLHCC (one out of seven) and seven cases of NHCC (seven out of 15) scored IS⩾5 (high invasive). Collectively, the SLHCC group showed lower IS scores than the NHCC group (*P*=0.02), suggesting that SLHCC is less invasive than NHCC.

### Identification of genes differentially expressed in SLHCC and NHCC

The distinct phenotypes displayed by SLHCC and NHCC implicate differences in biology. To gain a better understanding of the differences between SLHCC and NHCC, we made an attempt to profile gene expression patterns in these two types of HCC using cDNA microarray. By setting the threshold value of 2 (upregulation) and 0.5 (downregulation) for assessing the difference in the expression level between SLHCC and NHCC, 668 out of 8464 human genes (cDNA microarray, Biostar Genechip Inc., Shanghai, China) were differentially expressed (355 upregulated and 313 downregulated genes). The altered genes clustered into different subsets of cellular genes whose products are implicated in biological activities generally consistent with the physiology and pathology of SLHCC and NHCC. A partial list of the genes differentially expressed is shown in Table 2

**Table 2:** Parts of genes differentially expressed between SLHCC and NHCC

**GeneBank ID**	**Gene name**	**Function**	**Ratio (Cy5/Cy3)**
NM_031934	Homo sapiens RAB34, member RAS oncogene family (RAB34), mRNA	Cellular skeleton andmovement	0.224
NM_003177	Homo sapiens spleen tyrosine kinase (SYK), mRNA	Signal transduction	0.240
NM_014624	Homo sapiens S100 calcium-binding protein A6 (calcyclin) (S100A6), mRNA	Signal transduction	0.345
NM_022807	Homo sapiens small nuclear ribonucleoprotein polypeptide N (SNRPN), transcript variant 4,mRNA	Growth and development	0.389
NM_001168	Homo sapiens baculoviral IAP repeat-containing 5 (survivin) (BIRC5), mRNA	Cellular apoptosis	0.441
NM_175744	Homo sapiens ras homologue gene family, member C (ARHC), mRNA	Cellular skeleton andmovement	0.463
NM_002530	Homo sapiens neurotrophic tyrosine kinase, receptor, type 3 (NTRK3), mRNA	Growth and development	0.464
NM_001912	Homo sapiens cathepsin L (CTSL), mRNA	Matrix degrade	0.477
NM_000210	Homo sapiens integrin, alpha 6 (ITGA6), mRNA	Matrix degrade	0.493
NM_004394	Homo sapiens death-associated protein (DAP), mRNA	Cellular apoptosis	2.014
NM_001901	Homo sapiens connective tissue growth factor (CTGF), mRNA	Growth and development	2.026
NM_003445	Homo sapiens zinc finger protein 155 (pHZ-96) (ZNF155), mRNA	Signal transduction	2.095
NM_000389	Homo sapiens cyclin-dependent kinase inhibitor 1A (p21, Cip1) (CDKN1A), mRNA	Cellular apoptosis	2.292
NM_017675	Homo sapiens protocadherin LKC (PC-LKC), mRNA	Matrix degrade	2.358
AF220656	Homo sapiens apoptosis-associated nuclear protein PHLDA1 (PHLDA1) mRNA, partial cds	Cellular apoptosis	2.388
NM_001880	Homo sapiens activating transcription factor 2 (ATF2), mRNA	Cellular apoptosis	3.090
NM_001257	Homo sapiens cadherin 13, H-cadherin (heart) (CDH13), mRNA	Matrix degrade	4.60

. Complete information of the cDNA microarray can be accessed through our website. Collectively, the array data provide a genome-wide view of differences in gene expression between SLHCC and NHCC. Of particular interest among 668 genes that were differentially expressed is the *RhoC* gene, whose expression is higher in highly invasive NHCC than in less invasive SLHCC since RhoC expression has been implicated in tumour metastasis. We therefore decided to validate the array data by assessing the expression of RhoC at the level of message RNA and protein in the HCC samples.

### Expression of RhoC mRNA and protein

**Figure 2:**
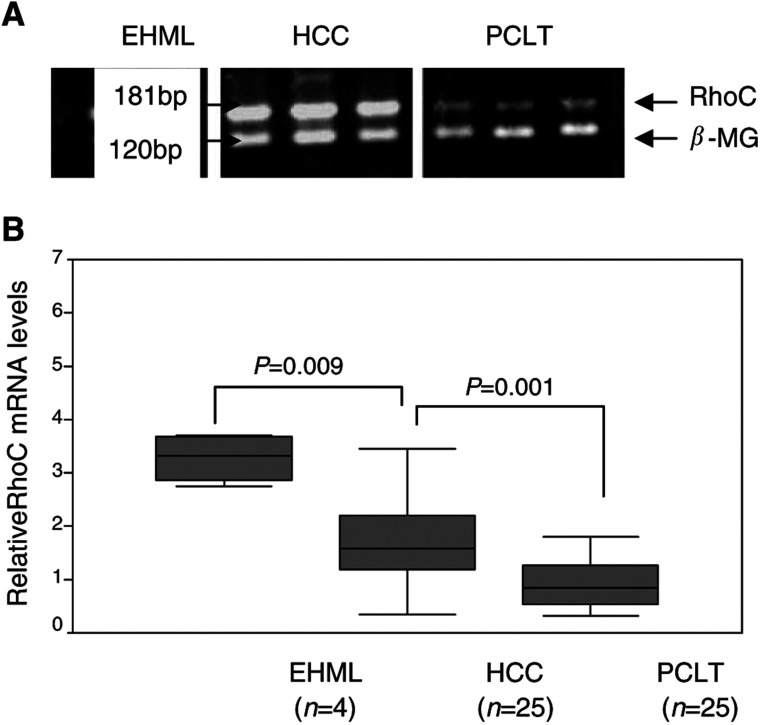
Detection of RhoC mRNA by RT–PCR. (**A**) PCR products were visualised by ethidium bromide staining. Eight selected samples from each group are shown: RhoC (181 bp); *β*-MG (120 bp); EHML: extrahepatic metastatic lesions; HCC: hepatocellular carcinoma tissues; PCLT: pericarcinomatous liver tissues. (**B**) The Mann–Whitney test was performed to compare the mRNA expression of RhoC between different groups; EHML showed a significantly higher mRNA expression level than HCC and the same between HCC and PCLT.

The expressions of RhoC mRNA and protein were detected in all HCC tissues and PCLT. However, the levels of both RhoC mRNA and protein are significantly higher in HCC tissues than in PCLT (*P*=0.001). Significantly, we detected substantially higher levels of both RhoC mRNA and protein in extrahepatic HCC tissues than those in intrahepatic HCC tissues (*P*=0.009 and 0.002, Figures 2

and 3

**Figure 3:**
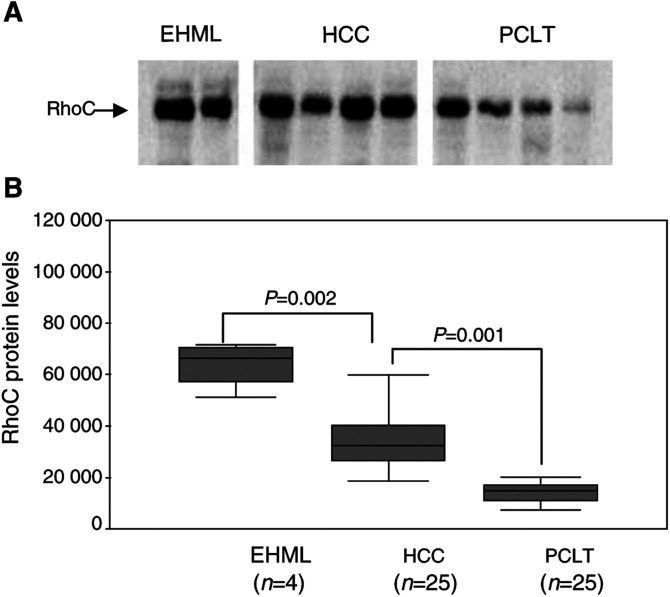
Western analysis of RhoC protein. (**A**) The protein expression levels were obtained on Kodak films and were quantified by densitometry. A total of 10 selected samples were shown. (**B**) The Mann–Whitney test showed significant differences between EHML and HCC, and also between HCC and PCLT.

). Statistical analyses revealed a strongly positive correlation (*r*=0.735, *P*<0.001, Figure 4

**Figure 4:**
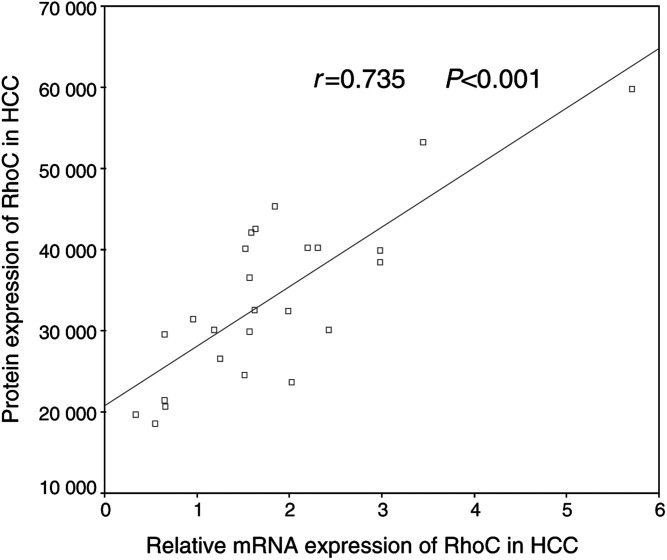
Correlation between mRNA and protein expression levels of RhoC in HCC was evaluated by Spearman's correlation coefficient. RhoC Protein levels were directly correlated with the levels of RhoC mRNA in HCC with adjusted *r*_s_=0.735, and two-tailed probability, *P*<0.001.

).

### Correlation between RhoC expression levels and clinicopathological parameters of HCC

The distribution pattern of RhoC was further analysed by dividing the expression levels of RhoC mRNA and protein in subgroups according to the clinicopathological parameters. Interestingly, the RhoC mRNA and protein expression levels in HCC with multinodes were significantly higher than those with one node (*P*=0.011 and 0.015). Furthermore, increased RhoC expression strongly correlates with vein invasion (*P*=0.006 and 0.030). A significant difference in RhoC expression was also evident between HCC with poor differentiation and those with well differentiation (*P*=0.004 and 0.002). There were no significant associations between expression of RhoC gene and other clinicopathological parameters such as sex, HBV infection, liver cirrhosis, tumour size and capsular condition (Table 3

**Table 3:** Relationship between expression level of RhoC and clinicopathological parameters

		***P*-value**	
**Variables**	**No. of patients**	**RhoC mRNA**	**RhoC protein**
*Sex*			
Male	22	0.675	0.452
Female	3		
			
*Liver cirrhosis*			
Present	14	0.584	0.622
Absent	11		
			
*Capsule formation*			
Present	13	0.785	0.744
Absent	12		
			
*Tumour node*			
⩾2	15	0.011	0.015
<2	10		
			
*Cell differentiation*			
I–II	9	0.004	0.002
III–IV	16		
			
*Vein invasion*			
Present	13	0.006	0.030
Absent	12		
			
*Extrahepatic metastasis*			
Present	5	0.103	0.077
Absent	20		
			
*Tumour size (cm)*			
>5	18	0.287	0.904
⩽5	7		

).

### Prognostic implications of RhoC expression

**Figure 5:**
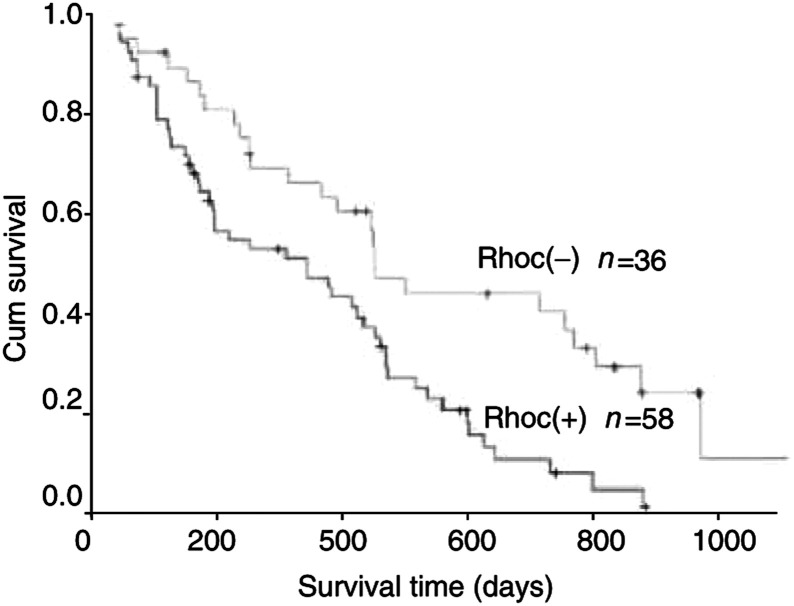
Kaplan–Meier survival curves for RhoC-positive expression group (*n*=58) and RhoC-negative expression group (*n*=36) based on the results of immunohistochemistry. HCC patients with RhoC-positive expression revealed a significantly poor prognosis than those with RhoC-negative expression (log-rank test, *P*=0.0031).

Kaplan–Meier survival curves for patients with HCC, categorised according to RhoC expression, are shown in Figure 5

. The mean survival of patients with RhoC-positive expression, which was determined by immunohistochemical staining (Figure 5), was significantly lower (356 days) than that of patients with RhoC-negative expression (579 days, *P*=0.0031), indicative of a positive correlation of RhoC expression with poor prognosis.

## DISCUSSION

Classification and prognosis of HCC has been traditionally based on the size of HCC; the large HCC is often considered as advance and unresectable. However, we have observed that there is one type of HCC, which is large in size but exhibits a lower invasive and metastatic potential. This type of HCC typically has just single node and we defined them SLHCC. On the contrary, there is another type of HCC in which invasion and metastasis often occur early despite their small size. This type of HCC, however, has usually more than one node. These observations of ours are consistent with the sixth TNM classification for HCC revised by UICC that the number of nodes, but not size of HCC, is the crucial factor for classification and prognosis of HCC (Poon and Fan, 2003). We routinely divide HCC, based on the distinct phenotype, into: SLHCC (diameter >5 cm, only one node), NHCC (node number ⩾2) and SHCC (diameter ⩽5 cm). Our clinical data indicate that NHCC displays a significantly greater potency in invasion and metastasis than SLHCC does. In keeping with the notion that cell differentiation status and vein invasion highly correlate with the invasion and metastasis of HCC (Liver Cancer Study Group of Japan, 1990; Farinati *et al*, 2000; Ohkubo *et al*, 2000; Utsunomiya *et al*, 2000), we found that SLHCC is generally better differentiated and has less vein invasion than NHCC. SLHCC is also prone to formulate capsulation. Together with the IS, our data indicate that NHCC in general has a greater propensity to undergo metastasis than SLHCC does.

The distinct phenotypes exhibited by SLHCC and NHCC would likely be attributable to differences in gene expression. Our microarray analyses indicate that it is indeed the case. Among 8464 human genes examined, a total of 668 genes are differentially expressed between SLHCC and NHCC. While further studies are required to examine how those genes might contribute to the pathology of HCC, the difference in gene expression may represent the molecular basis underlying the distinct phenotypes exhibited by NHCC and SLHCC.

Owing to its role in cytoskeletal reorganisation, in focal adhesion contacts (Nobes and Hall, 1995; Kimura *et al*, 1996; Leung *et al*, 1996) and in tumour invasion (Clark *et al*, 2000), RhoC was chosen from the pool of 668 differentially expressed genes for further analysis of the possibility that increased RhoC expression might correlate with HCC metastasis. Three different approaches, RT–PCR to measure mRNA levels, Western and immunohistochemical staining to determine protein levels, were employed to verify the expression of RhoC. Our data indicate that the expression of RhoC was significantly higher in HCC tissues than that in the corresponding PCLT. Furthermore, increased expression of RhoC in HCC seemed to correlate positively with poor cell differentiation and with tumour vein invasion. It is well documented that HCC often undergo dedifferentiation (from well-differentiated to poorly differentiated) during multistep tumour progression (Mise *et al*, 1998), and that some HCC acquire metastatic potential during this progression, resulting in vein invasion (Hui *et al*, 1999). Our results suggest that increased expression of RhoC is closely associated with the later events in liver carcinogenesis.

Our present study also showed that extrahepatic metastasis of HCC expressed significantly higher RhoC levels than intrahepatic HCC, and elevated expression of RhoC positively correlated with vein invasion, and the number of tumour nodes. Similar observations have been reported in other types of tumours. For instance, overexpression of RhoC was found in metastatic lesions of inflammatory breast cancer and pancreatic ductal adenocarcinoma (Suwa *et al*, 1998; Fritz *et al*, 2002; Kleer *et al*, 2002), and upregulation was associated with tumour progression in ovarian carcinoma (Horiuchi *et al*, 2003). While the molecular mechanism by which RhoC facilitates tumour metastasis remains to be determined, it is possible that increased expression of RhoC could result in: (1) disruption of cell polarity, which plays an important role in the epithelial–mesenchymal transition observed in more aggressive tumours (Zondag *et al*, 2000); (2) contribution to the loss of adherens junctions (Braga *et al*, 2000); (3) increase motility and ability to remould the extra-cellular matrix, which is required for tumour cells to become locally invasive (Khanna *et al*, 2001); and (4) increase in angiogenic factors that would result in promotion of vascularisation in tumour and increase the likelihood of tumour cell entering the bloodstream (van Golen *et al*, 2000).

Our data suggest a possibility that RhoC expression could be used as a potential prognostic marker for HCC patients. To test this possibility, anti-RhoC immunohistochemical staining was performed on 94 cases of HCC that included the 25 cases mentioned above. Owing to the lower detection sensitivity of the immunohistochemistry method, some of the 94 cases of HCC showed negative staining of RhoC, despite the fact that RhoC protein can be detected in all 25 cases of HCC with Western blotting. Nevertheless, when we divided the total cases of HCC into either a RhoC-positive or -negative group, the RhoC-negative HCC patients in general had a better prognosis than the RhoC-positive HCC patients. Together, our data strongly suggest that increased RhoC expression in HCC correlates with a poor prognosis.
